# Modeling regionalized volumetric differences in protein-ligand binding cavities

**DOI:** 10.1186/1477-5956-10-S1-S6

**Published:** 2012-06-21

**Authors:** Brian Y Chen, Soutir Bandyopadhyay

**Affiliations:** 1 Department of Computer Science and Engineering, Lehigh University, Bethlehem, PA, USA; 2 Department of Mathematics, Lehigh University, Bethlehem, PA, USA

## Abstract

Identifying elements of protein structures that create differences in protein-ligand binding specificity is an essential method for explaining the molecular mechanisms underlying preferential binding. In some cases, influential mechanisms can be visually identified by experts in structural biology, but subtler mechanisms, whose significance may only be apparent from the analysis of many structures, are harder to find. To assist this process, we present a geometric algorithm and two statistical models for identifying significant structural differences in protein-ligand binding cavities. We demonstrate these methods in an analysis of sequentially nonredundant structural representatives of the canonical serine proteases and the enolase superfamily. Here, we observed that statistically significant structural variations identified experimentally established determinants of specificity. We also observed that an analysis of individual regions inside cavities can reveal areas where small differences in shape can correspond to differences in specificity.

## Background

Engineering or reverse engineering the molecular mechanisms that underlie specificity in protein-ligand binding is a crucial challenge in many fields. Understanding these mechanisms can explain, for example, why resistance occurs against certain drugs and not others [1], how we can mutate proteins to alter binding preferences [2], or how preferential binding in a few crucial molecules can control the organization of molecular and cellular environments [3]. The heart of this challenge lies in the fact that the mechanisms driving specificity are a product of multiple interacting components, such as amino acids [1] or cavity regions [4]. Fortunately, when the components involved in the mechanism are unknown, molecular structures can suggest testable possibilities, based on spatial proximity and biophysical principles.

One such principle relates to the shape of ligand binding cavities from families of closely related proteins. In such families, regions where cavities vary may cause differing substrates to bind. Similar regions might bind a molecular fragment that is common to substrates acted on by the entire family. This principle has been observed frequently, such as in the serine proteases, where binding cavities vary in size to better accommodate differently sized substrates [5], and in the enolase superfamily, where varying arrangements of amino acids around a common scaffold enable related but distinct reactions to be catalyzed [6-8]. Structural variations of this kind can sometimes be identified by visual inspection, but when many exist, or when they are very subtle, it can be unclear whether the variations found are significant enough to test experimentally as potential specificity determinants. Visual inspection is even harder when many structures must be considered, or when the flexibility of proteins must be taken into account.

**Figure 1:**
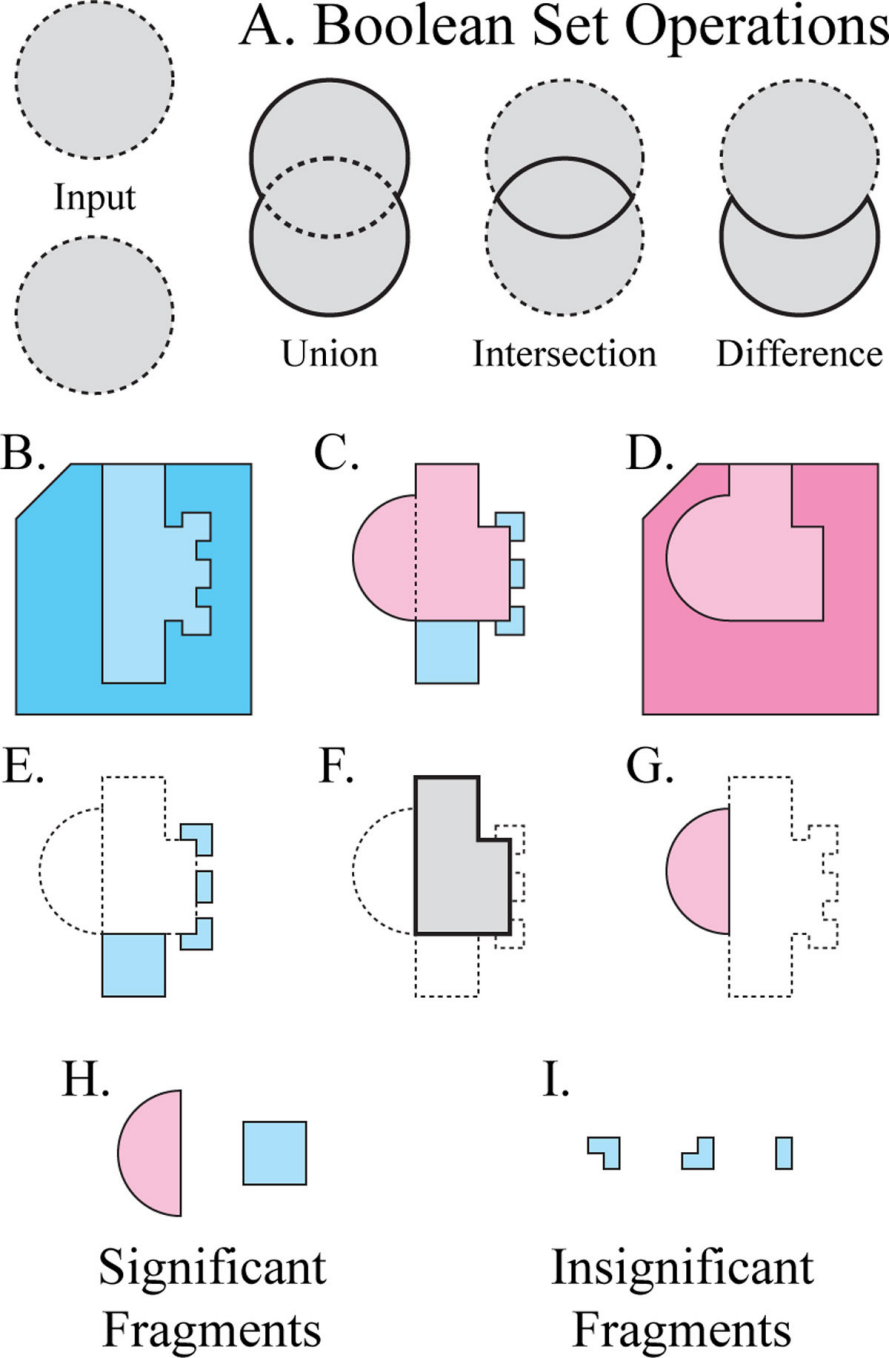
**Isolating significant cavity regions with Boolean Set operations**. A. A diagram of Boolean Set operations, showing the borders of input regions (dotted) and output (solid), in grey. B,D) Polygons representing regions occupied by protein x (blue) and protein Y (red), their molecular surfaces (black lines), and their binding cavities x (light blue) and y (light red). C) Superimposition of x and y, based on a whole structure alignment of X and Y. In E, F, and G, the superposition of x and y is depicted as dotted lines. Regions in solid lines were computed with Boolean set operations. E) The difference of x and y. F) The intersection of x and y. G) The difference of y and x. H) Statistically significant fragments from E and G. I) Statistically insignificant fragments from E and G.

To assist in this challenge, this paper presents a computational method and two statistical models for evaluating whether structural variations are significant enough to potentially alter specificity. Our methods leverage techniques for representing protein structures and cavities as geometric solids and for comparing them with Boolean Set operations (Figure 1). From this starting point, we describe a new capability for separating contiguous regions, called *fragments*, that lie within one cavity and not within another (e.g. Figure 1h,i). A fragment is thus one of possibly several shape differences between two cavities, and it may create a difference in binding specificity. We hypothesize that most fragments, which are very small, do not influence specificity, and that fragments that are unusually large may be more influential.

Seeking to automatically isolate potential influences on specificity along these principles, we introduce two statistical models for evaluating fragments. The first model, referred to below as the "standard model", represents the volume of fragments that occur between binding cavities of proteins with identical specificity. This approach can identify fragments that are too large to be consistent with cavities having identical specificity, and, in our experimental results, we observed that it can thus isolate regions in cavities that influence specificity.

The second model, the "regionalized model", represents fragments between training set cavities that lie within a user-defined region. The regionalized model thus redefines statistical significance based on local differences in the training set: Small but statistically significant fragments can be isolated in regions where training set cavities hardly differ, while equally sized fragments might be statistically insignificant in regions where training set cavities differ wildly. In ligand design applications, where ligand skeletons can be altered at limited sites, the regionalized model might reveal local cavity differences that point to the design of a more selective inhibitor. Below, we demonstrate the capabilities of these models on binding cavities in the serine proteases and the enolase superfamily. Together, these models represent a comprehensive statistical framework for analyzing fragments between similar cavities.

### Related work

The solid representations of protein structures and binding cavities used in this work differ considerably from typical comparison algorithms, which typically employ point-based and surface-based representations. Point-based representations encode atoms in protein structures using points in three dimensions [9-13], matrices of distances between points [14], and nodes in geometric graphs [15,16]. These representations are traditionally applied to rigidly superpose and align whole protein structures, but, more recently, flexible methods [17] have also emerged. A second type of point-based representation is specialized for the comparison of functional sites, using motifs in three dimensions that encode atoms in catalytic sites [18-20], evolutionarily significant amino acids [21], "pseudo-centers" representing protein-ligand interactions [22], and pseudoatoms representing amino acid sidechains [23]. Point-based methods exhibit extreme efficiency, enabling them to rapidly search for evolutionarily remote homologs [18,19,24] in large databases of protein structure [25], but they are not intended for isolating variations in empty cavity regions, like the methods presented here.

Surface-based representations employ closed surfaces or surface patches to represent or approximate solvent-accessible shape [26,27]. These representations are built from triangular meshes [28,29], alpha shapes [30-32], three dimensional grids [33], and spherical harmonics [34-36]. In some cases surface representations have been applied for the comparison of protein structures [28,29] and electrostatic potentials [37], as well as in hybrid representations that combine point-based and surface-based information [38], but they have had widest application in the identification of cavities and hot spots [39] in protein surfaces [30,40-42]. While surface-based methods identify and compare surface cavities, the work described here offers the new capability of isolating individual variations within cavities.

Volume-based concepts can be found in algorithms that compute molecular surfaces [43-47], in many techniques for identifying protein-ligand binding pockets (e.g. [40,48-50]), and for representing electrostatic isopotentials [12]. Throughout, volume-based techniques have more visibly been applied for the visualization of protein structures, but not for their comparison. For example, slab-based visualizations, which render a protein in cross section, are a fixture of protein structure visualization tools like Pymol [51] and Rasmol [52]. Slab-based visualization uses rendering parameters such as the view frustrum and Goldfeather-like algorithms [53] to draw the slab, rather than explicitly computing the geometry of the region defined by the cross section. In contrast to existing work, techniques using Boolean Set operations to identify influences on specificity [54-56], are distinct in both methodology and application.

Statistical modeling plays a critical role in the unsupervised comparison of protein structures, especially in the identification of geometrically similar catalytic sites at remote evolutionary distances. In that application, statistical modeling enables the computation of data-specific thresholds to identify catalytic sites that are improbably similar, and thus potential markers of functional similarity. Several independent results, using Gaussian mixture models [19], extreme value distributions [57], nonparametric models [18], and empirical models [31,58], have observed that statistically significant geometric similarity is an accurate marker of similar functional sites. In contrast to existing work, we introduce a new application for statistical modeling by first paraphrasing our standard model for cavity variation, described earlier [55], and then extending that model to represent local variations within a user defined region.

## Methods

In earlier work, we demonstrated that individual fragments could be automatically separated and that a statistical model of fragment volume could be used to identify fragments of unusually large (e.g. statistically significant) size [54]. We paraphrase this work here, for completeness. We have since extended this work by showing that we can restrict the training of our model on structural variations within a user defined cube. This regionalized approach enables our statistical model to vary it's significance thresholds based on the local variations of training set cavities in the region defined by the user.

### Identifying fragments

**Figure 2:**
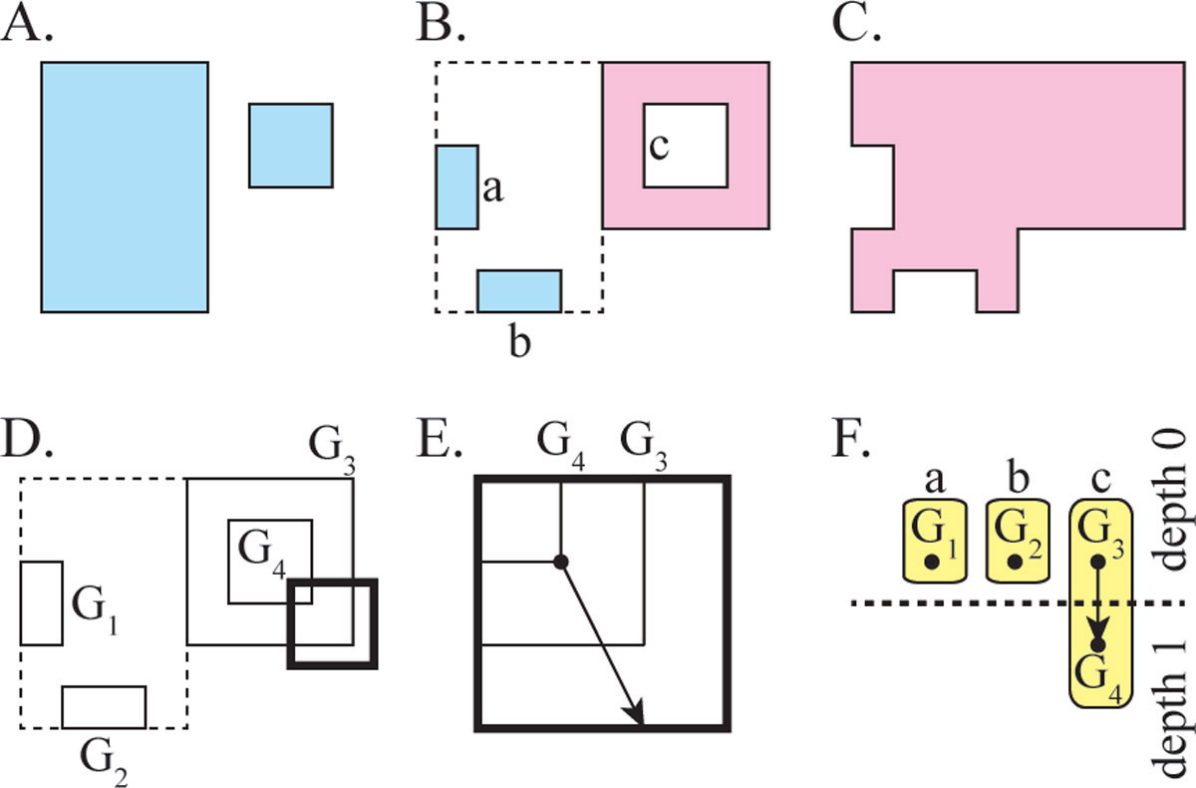
**Computing fragments**. Input cavity *A *(light blue,A) and *B *(light red,C). Fragments derived from *AB *and *BA*, labeled with lower case letters, original cavities outlined in dotted lines (B). Fragments translated into connected components (solid lines, labeled *G_1_*-*G_4_*), original cavities outlined in dotted lines (D). A ray (arrow) from a corner of *G_4_*, drawn from the enlarged perspective of the dark box in D (E). The directed graph, *H*, with nodes *G*_1 _− *G*_4_, connected based on containment. The dotted line separates nodes of different depths. Yellow ovals indicate subgraphs relating to the same fragment, labeled with lower case letters (F). Cavity A is shown atypically, in a disconnected manner, to illustrate the algorithm.

We begin with two geometric solids, *A *(Figure 2a) and *B *(Figure 2c), representing cavities from aligned protein structures. Using Boolean Set operations, we compute *AB*, the region inside *A *and not inside *B*, as well as *BA*, the region inside *B *and not *A*. Like *A *and *B*, *AB *and *BA *are geometric solids represented by closed triangular meshes. Fragments from *AB *and *BA *are, together, referred to as the set of fragments relating to cavities *A *and *B *(Figure 2b).

First, we translate the triangular mesh of *AB *and *BA *into a graph *G*, mapping corners to graph nodes, and triangle edges to graph edges. Since *G *is likely to have several connected components, we separate each connected component into an individual graph *G_i _*(Figure 2d). This can be accomplished through depth first search in linear time [59]. Each connected component does not necessarily represent an individual fragment, because fragments occasionally contain interior voids, as illustrated in Figure 2b, that are composed of multiple disconnected components.

Next, we determine which connected components reside within another connected component. This is accomplished with ray casting (Figure 2e). For each component *G_i_*, a ray, beginning at one point on *G_i _*is pointed in a random direction, and the number of intersections with other components is counted. If the ray intersects another component *G_j _*an even number of times, then we say that *G_i _*is not inside *G_j_*. Alternately, if the ray intersects *G_j _*an odd number of times, then we say that *G_i _*is inside *G_j_*. For all pairs *G_i _*and *G_j_*, we determine which contains the other.

Next, we represent the pattern of containment as a directed acyclic graph *H *(Figure 2f), where each node represents a graph *G_i_*, and an edge from *G_i _*to *G_j _*indicates that *G_j _*is within *G_i _*according to the test above. This graph is redundant, because all nested fragments pass the test, but only some nested fragments are part of the same fragment. Fortunately, using the topology of *H*, we can determine which *G_i _*are part of the same fragment: First, we identify subgraphs $G_{i_{k}}$ that are not contained inside any other graph, because their in-degree is zero. From each $G_{i_{k}}$, we perform a depth first search and assign an integer depth *d *to each *G_i _*considered. Since *H *is an acyclic graph, some *G_i _*may be visited more than once. In these cases, if the number of edges traversed from the originating $G_{i_{j}}$ to the current *G_i _*is greater than the depth assigned already, *d *is reassigned the larger value. This reassignment process determines the number of times each graph *G_i _*is nested within the entire group of connected components, since the largest possible depth reflects the actual number of times one graph is nested inside the others.

Finally, we separate *H *into subgraphs. Each *G_i _*with an even depth *d *is an exterior surface for one fragment. Based on the topology of *H*, each *G_i _*with an odd depth *d *resides inside an exterior surface with even depth equal to *d *− 1. Thus, we can associate the graphs *G_i _*into groups that are all part of the same fragment, and output the fragment. This correctly separates fragments of arbitrary nesting.

### The standard model of fragment volume

Our statistical model is based on a hypothesis testing framework that detects fragments with volume large enough to be statistically significant, i.e. unlikely to occur by random chance. The underlying assumption of our model is that fragments derived from cavities with no difference in specificity will have *small *volumes related to incidental and functionally irrelevant structural variation. Alternatively, if there exists a structural variation in one cavity large enough to create a steric influence on specificity, then the fragment generated by the variation between the cavities will have *unusually large *volume. Thus, for a query fragment *F *, based on cavities *A *and *B*, our null hypothesis asserts that the volume of *F*, *v*(*F *), is *small*. The alternative hypothesis asserts that *v*(*F*) is *unusually large*. Since they are logical complements, exactly one of these hypotheses can hold for any fragment *F*.

We test the null hypothesis by first assuming that it holds for *F*, and then estimating the probability *p *of randomly observing another fragment *F*', with volume *v*(*F*') ≥ *v*(*F*). If the probability of randomly observing another fragment with larger volume is improbably low, typically below 0.05, then it is hard to continue assuming that *F *is small. In this circumstance, the null hypothesis is rejected as improbable, leaving us to favor the alternative hypothesis, that *F *is large. The biological interpretation of this decision follows from our underlying assumption: *F *is unusually large, and may thus be a structural variation in either *A *or *B *that creates a steric influence on specificity. This statement is a prediction based on quantified evidence, not a statement of fact.

In order to perform this prediction, we must estimate the probability *p*, which requires us to first train the statistical model. Training begins with aligned cavities from the training sets described in Section. First, we separate the fragments generated between all pairs of cavities using the method described in Section. Using the Surveyor's Formula [60], which provides a rapid and very accurate estimation of volume in a closed surface, we compute the volume of each fragment. These data are represented in a frequency distribution *D *(See Figure 5A). The shape of *D *closely fits a log-normal distribution, as seen in Section.

**Figure 3:**
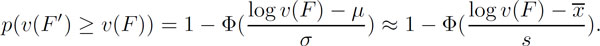
**Estimating *p*-values**. Estimating the probability *p *of observing a fragment *F*', with volume *v*(*F*') ≥ *v*(*F*), using the mean ($\bar{x}$) and variance (*s*) of the distribution of the log-transformed sample values to estimate *µ *and *σ*. Φ is the cumulative distribution function of the standard normal distribution.

Since *D *fits *log-normal*(*µ*, *σ*), we can use the log-normal distribution to smoothly estimate the probability *p *of observing any a fragment *F*', with volume greater than or equal to the volume of our query fragment *v*(*F*). This estimation occurs when we realize that the mean *µ *and the variance *σ *of the log-normal distribution are unknown: we estimate *µ *and *σ *with the mean $\bar{x}$ and variance *s *from the distribution of log-transformed values of *D*. We can thus estimate *p *using the Equation in Figure 3. *p *is the proportion of the volume under the log-normal curve to the right of *v*(*F*), relative to the total volume under the curve (*x *≥ 0).

Fitting the log-normal function to *D *enables this probability to be estimated without the discretizing effect of the training data. Also, assuming that the log-normal distribution is a sufficiently accurate estimation of the underlying probability density function, we can extrapolate the probability beyond the largest volume observed in our training data. Such extrapolation would not be possible using nonparametric models, which have finite support. The accuracy of this extrapolation is illustrated in our results.

Having trained our statistical model on fragments derived from cavities with identical binding specificities, we hypothesize that our statistical model will behave as follows: Fragments generated between a cavity binding preferences similar to those of the training set and a cavity with different binding preferences are be expected to have a statistically significant fragment, if there exists a steric influence on specificity. Likewise, for two cavities having the same binding preferences as the training set, fragments generated between them are not expected to be statistically significant. We test this hypothesis in our experimental results.

### The regionalized model of fragment volume

Our regionalized model has the same theoretical foundation as the standard model, with some critical differences. Like the standard model, it is also based on a hypothesis testing framework for detecting improbably large fragments within a user-defined cube *g*. The null hypothesis asserts that a given fragment *F *within *g *has a *small *volume, and the alternative hypothesis asserts that *F *has an *unusually large *volume.

The fragments used to train the regionalized model are generated in the same way as in the standard model, except that the Boolean intersection of every fragment and *g *is computed after all fragments are generated. This extra step results in the elimination of many fragments that do not intersect *g*, and a reduction in the volume for fragments that are partially contained in *g*.

Like the standard model, the distribution *D *of volumes from fragments regionalized to *g *in this way are fit to a log-normal distribution. Given a fragment *F*, Equation 3 allows us to estimate the probability of observing a fragment with volume equal to or greater than those that typically occur inside *g *between training set cavities. This approach functions like that of the standard model, with one special case: It may be that *g *intersects no training set cavities. In such cases, when asked to estimate the *p*-value of a fragment in *g*, we assert categorically that they are significant, because the fragment relates to a difference in shape that is not reflected by any training set cavity in the same region.

### Data set construction

#### Protein families

The serine protease and the enolase superfamilies were selected for demonstrating our statistical models because several sequentially nonredundant structures exist for both superfamilies. Each superfamily contained at least three subfamilies with distinct binding preferences and at least two nonredundant structural representatives in each subfamily.

Serine proteases catalyze the hydrolysis of specific peptide bonds by recognizing neighboring amino acids with specificity subsites numbered *S*4, *S*3, . . . *S*1, *S*1^'^, *S*2^'^, . . . , *S*4^'^. Each subsite preferentially binds one amino acid before or after the hydrolyzed bond between *S*1 and *S*1^'^. Our demonstration, on three subfamilies, focuses on the *S1 *subsite, which binds aromatics in chymotrypsins [61], positively charged amino acids in trypsins [62], and small hydrophobics in elastases [63].

Members of the enolase superfamily catalyze a variety of reactions that involve the abstraction of a proton from a carbon adjacent to a carboxylic acid [6]. Assisted by an N-terminal "capping domain" [64], amino acids at the C-terminal ends of beta sheets in a conserved TIM-barrel act as acid/base catalysts to facilitate several different reactions [6]. Our demonstration, on three subfamilies, is focused on the primary catalytic site, which facilitates the dehydration of 2-phospho-D-glycerate to phosphoenolpyruvate in enolase, [65], the conversion of (R)-mandelate to and from (S)-mandelate [66] in mandelate racemase, and reciprocal cycloisomerization of cis,cis-muconate and muconolactone in muconate-lactonizing enzyme [6].

#### Selection

**Figure 4:**
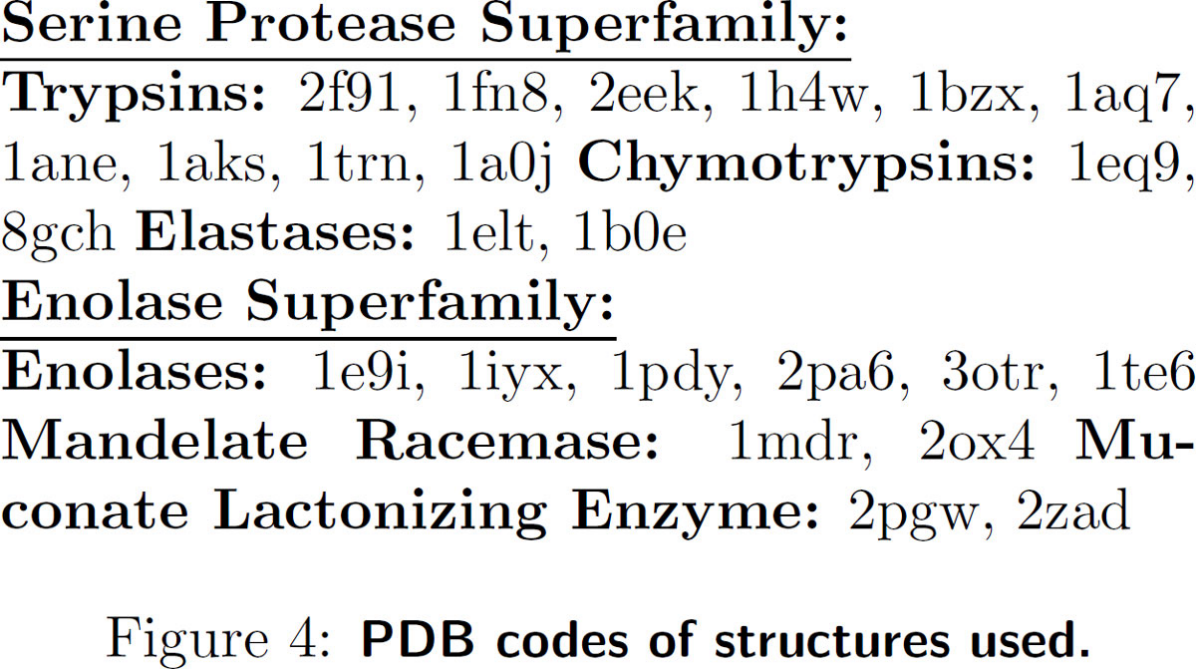
**PDB codes of structures used**.

The Protein DataBank (PDB - 6.21.2011) [25] contains 676 Serine proteases from chymotrypsin, trypsin, and elastase subfamilies and 66 enolase superfamily structures from enolase, mandelate racemase, and muconate cycloisomerase subfamiles. From each set, we removed mutant and partially ordered structures. Because enolases have open and closed conformations, all closed or partially closed structures were removed. Next, structures with greater than 90% sequence identity were removed, with preference for structures associated with publications, resulting in 14 serine protease and 10 enolase structures (Figure 4). Within these structures, ions, waters, and other non-protein atoms were removed. Since hydrogens were unavailable in all structures, all hydrogens were removed for uniformity. Atypical amino acids (e.g. selenomethionines) were not removed.

#### Alignment

Ska [13], an algorithm for whole-protein structure alignment, was used to align all serine protease structures to bovine gamma-chymotrypsin (pdb code: 8gch), and all enolase superfamily structures to mandelate racemase from pseudomonas putida (pdb code: 1mdr). Since proteins in these datasets have identical folds, alignments to a different structure has little effect: This was observed earlier [54], where cavity comparisons, recomputed with the same method, generated identical results. Beginning with this alignment, solid geometric representations of binding cavities were generated with a method described earlier [54], based on cavities defined in SCREEN [40].

#### Cavity preparation

Solid geometric representations of binding cavities were generated with a method described earlier [54], and paraphrased here for convenience. First, for each of the aligned structures, using GRASP2 [12], which applies the classical rolling-probe technique [27], we compute a molecular surface, with a water-sized probe of radius 1.4 Å, and an "envelope surface" with a probe of radius 5.0 Å. The radius of the larger probe is based on an external cavity boundary used in SCREEN [40]. Second, spheres with a radius of 5 Å, are centered at atoms bound in the binding sites of 8gch and 1mdr. In the case of 8gch, these are a tryptophan amino acid and five waters in the S1 subsite [67], and in the case of 1mdr, these are are the heavy atoms of a bound atrolactic acid molecule [68]. Since all structures are aligned to either 8gch or 1mdr, the 5 Åspheres completely fill the now-superposed binding sites of all structures in both sets. Third, we compute the Boolean union of the spheres in 8gch, and, separately, the Boolean union for spheres in 1mdr. The remainder of the procedure is performed identically for each member of the serine protease set and the enolase set, using the corresponding sphere union: We compute the boolean difference between the sphere union and the molecular surface, and then the intersection between the resulting difference region and the molecular envelope. The result is a geometric solid representing the binding cavity.

## Results

### Validating the standard model

**Figure 5:**
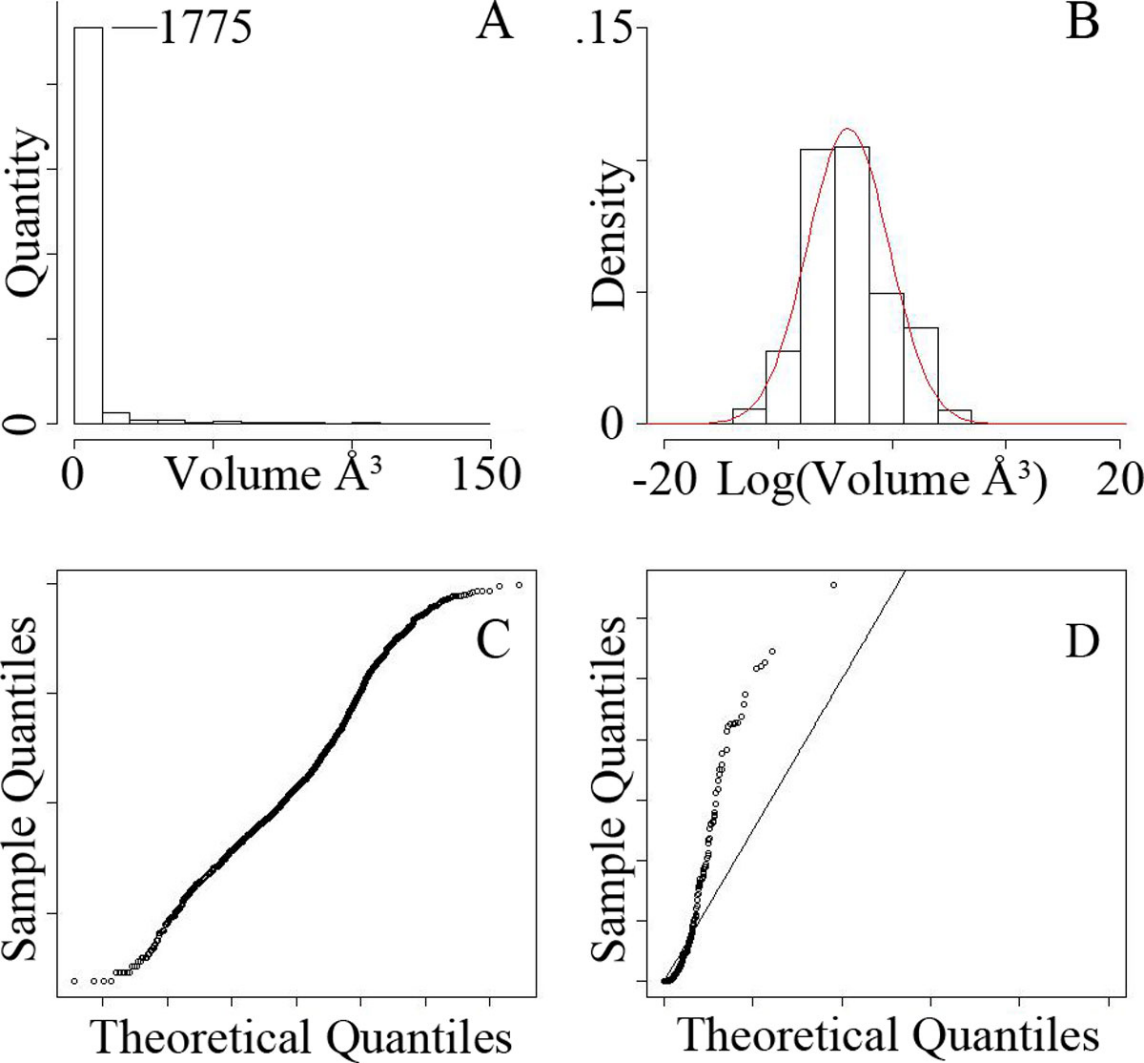
**Unified statistical model data**. Histogram counting of the number of fragments between pairs of trypsin cavities, in volume bins (A), and in log(Volume) bins plotted against the best fitting Gaussian (B). Quantile-quantile plots of trypsin fragment volumes relative to the best fitting Gaussian (C) and Gamma distributions (D).

We constructed a statistical model based on all trypsin cavities and a second based on all enolase cavities. The distribution of fragment volumes between trypsin and enolase cavities is illustrated in Figure 5A. Fragments with volumes near zero dominated, though both distributions exhibited a positive tail. Seeking the best fitting parametric model, we tested gamma, Weibull, Pareto, generalized extreme value, and log-normal distributions.

In both Trypsin and Enolase sets, we observed that log-normal distributions fit the observed data best. This is apparent in part when considering how well the Gaussian distribution fits the log of the fragment volumes (Figure 5A), but even more so when considering the quantile-quantile (q-q) plots comparing the log of observed fragment volumes versus a Gaussian distribution (Figure 5C). Other distributions considered led to poorer q-q plots: The second best, in both cases was the gamma distribution (Figure 5D). Enolase plots (not shown) were similar in overall shape, and supported the same conclusions. All plots are available here: http://www.cse.lehigh.edu/~chen/papers/BIBM2011.

### Calculating fragment significance

**Figure 6:**
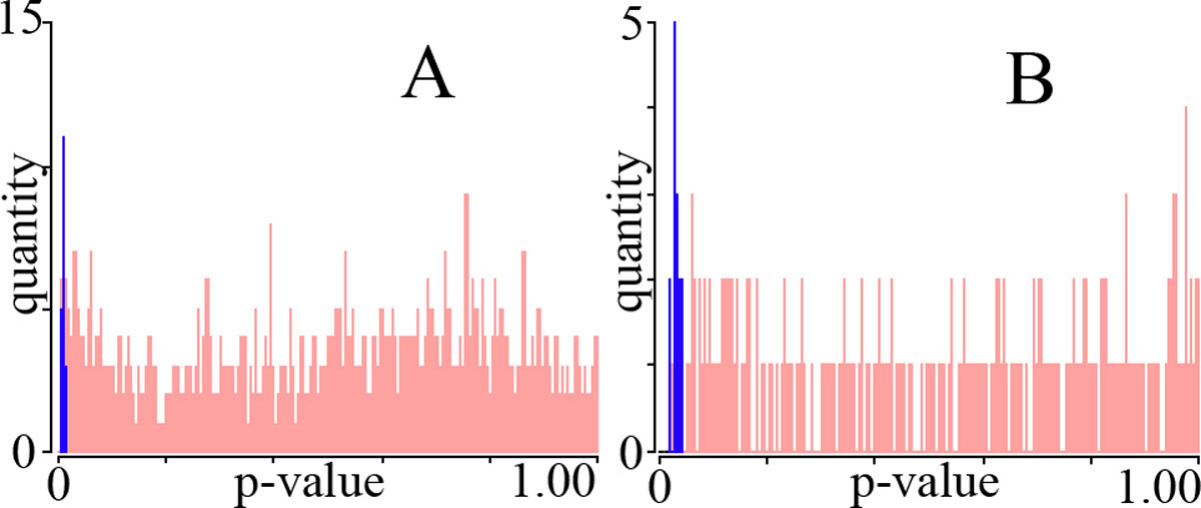
**P-values of serine protease and enolase fragments**. Histograms of fragments in *p*-value bins, depicting fragments from cavities with different specificities (dark blue) and fragments from cavities with similar specificities (light red), in proteins from serine protease (A) and enolase (B) superfamilies.

We calculated the statistical significance of fragments that exist between cavities with different binding specificities, in a leave-one-out manner: First, the statistical model was trained on all but one trypsin or enolase cavity. With the remaining trypsin or enolase cavity and each of the non-trypsin or non-enolase cavities, we determined the largest fragment, and measured its *p*-value. This process was repeated once each trypsin and enolase cavity, producing 40 trypsin fragments and 36 enolase fragments (Figure 6, dark blue).

We also calculated the statistical significance of fragments that exist between cavities with the same binding specificities. In a leave-two-out experiment, we first trained the statistical model with all but two trypsins or elastases. With the remaining two trypsins or elastases we computed the *p*-value of all fragments. This process was repeated for every combination of two members in the trypsin and enolase sets, producing 1893 trypsin fragments and 340 enolase fragments (Figure 6, light red).

The largest fragments from cavities with different binding preferences were always statistically significant, following the standard 0.05 threshold of statistical significance. By the same standard, fragments from cavities with identical binding preferences were rarely significant, exhibiting widely distributed *p*-values.

### Verifying fragment function

**Figure 7:**
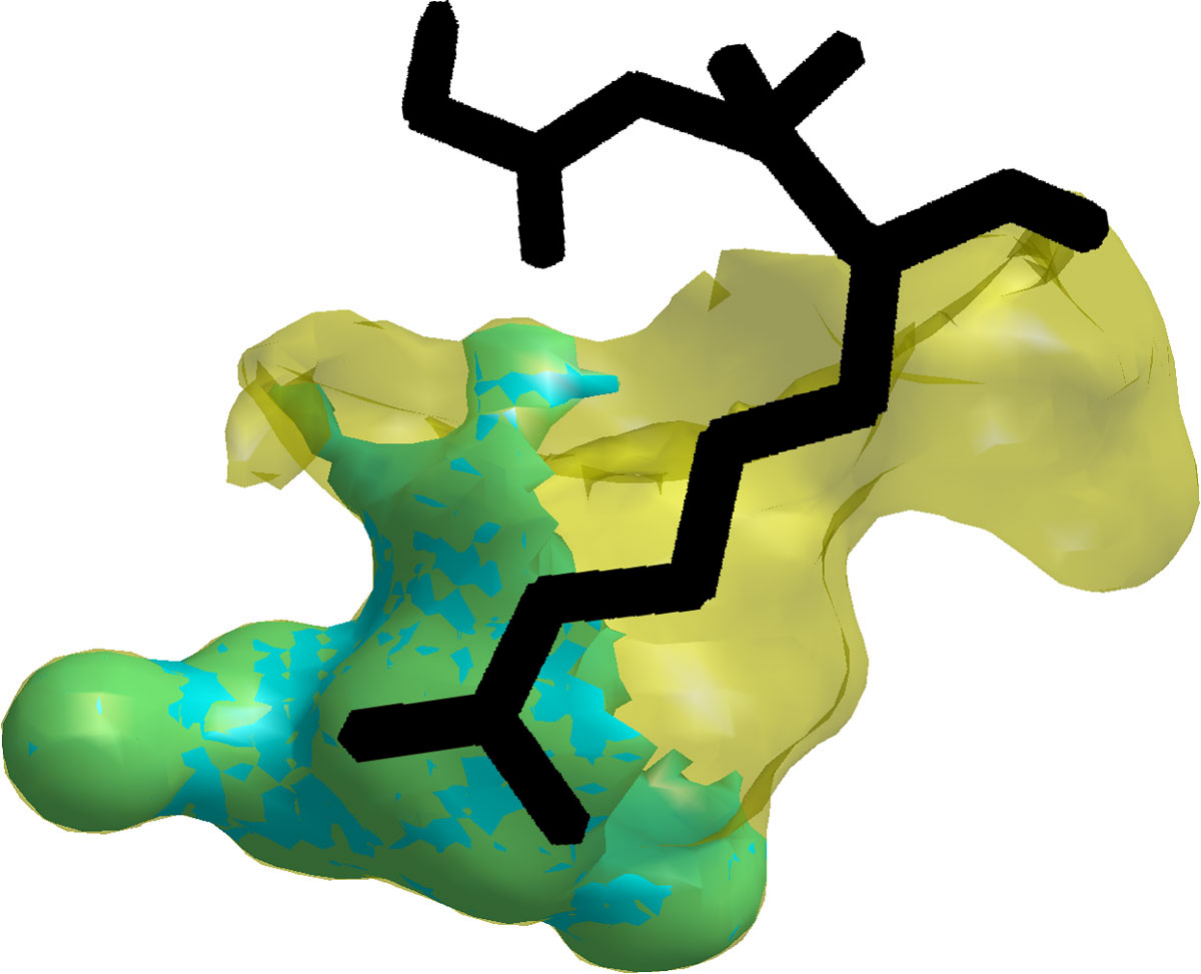
**An influential fragment**. S1 specificity site of Atlantic salmon trypsin (transparent, yellow). Statistically significant fragment within the trypsin cavity and not within the S1 specificity site of porcine pancreatic elastase (opaque, teal). Gly-Ala-Arg peptide modeled from Fusarium oxysporum trypsin (black sticks).

Statistically significant fragments identified several variations in cavity shape that influence binding preferences. One example, illustrated in Figure 7 depicts a statistically significant fragment that is within the S1 specificity site of Atlantic salmon trypsin (pdb reference: 1a0j) and not within the S1 specificity site of porcine pancreatic elastase (pdb reference: 1b0e). The fragment occupies a volume of 144 Å^3^, and is the largest of several differences between these cavities. The position of the fragment highlights a region in the trypsin cavity that extends deeper than the elastase cavity. This region is essential for accommodating the longer, positively charged substrates preferred by trypsins [62]. A modeled Gly-Ala-Arg peptide illustrates this point in Figure 7. Much like this example, similar significant fragments could be found between all trypsin and elastase cavities, as well as between trypsin and chymotrypsin cavities. A second class of related effects were observed in enolase cavities, where sidechains protruding from different parts of the conserved beta-barrel scaffold created differences in cavity shape that accommodate different catalytic reactions.

### Validating the regionalized statistical model

To establish the best fitting model, we tested gamma, Weibull, Pareto, generalized extreme value, and log-normal distributions as parametric models of the distribution of fragment volumes inside a user defined region *g*. To test a range of regions, we generated lattices of 252 and 125 cubes, with side-lengths of 5 Å, that generously surrounded the aligned trypsin cavities and enolase cavities, respectively. Due mainly to the wide margins used to surround the training cavities, 229 and 103 cubes surrounding the trypsin and enolase training sets, respectively, did not intersect with fragments from the training set.

**Figure 8:**
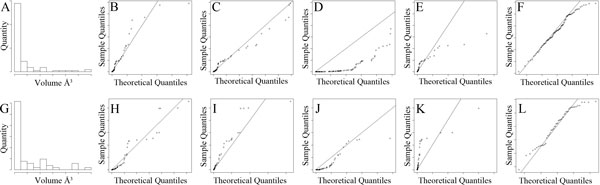
**Fitting the regional log-normal model**. Histogram of regional fragment volumes between pairs of trypsin (A) and enolase (G) cavities. Quantile-quantile plots of regional fragment volumes (sample quantiles) relative to the best fitting gamma, Weibull, Pareto, generalized extreme value, and log-normal distributions (theoretical quantiles) among trypsin (B-F) and enolase (H-L) fragments.

Five cubes surrounding the trypsin cavities and a second five surrounding the enolase cavities were randomly selected from the cubes that did contain fragments. Using the fragments inside these cavities, we generated a distribution of fragment volumes for each cube, and computed the best fitting gamma, Weibull, Pareto, generalized extreme value, and log-normal distributions. In every case, quantile-quantile plots indicated that the log-normal distribution was a more accurate model for the data. This is apparent in Figure 8, which illustrates q-q plots for one trypsin and one enolase cube.

### Statistically significant regional fragments influence specificity

Beginning with the lattices generated in the previous section, we trained our regional statistical models on the cavities of nine of the ten trypsins (all but human trypsin, pdb: 1h4w) and five of the six enolases (all but enolase from Toxoplasma gondii, pdb: 3otr). This created regional models corresponding to 252 trypsin lattice cubes and 125 enolase latice cubes, though most were trivial, as mentioned earlier. In this case, lattices simulate a wide range of possible user inputs. The cavities of 1h4w and 3otr were used for fragment generation with the non-trypsin serine proteases and the non-enolase Enolase superfamily members, respectively. The intersection of these fragments with each of the lattice cubes was determined, and their statistical significance within each regional model was estimated.

Every statistically significant fragment identified by a regional model was a part of a larger difference in cavity structure that is related to a difference in binding specificity. Categorically significant fragments plainly distinguished the major structural differences between cavities with different binding preferences. Among enolases, the variations between the mandelate racemases and 3otr exhibited several regions of this nature, occupying approximately 27 Å^3^. These differences in cavity shape were caused by the different placement of amino acids surrounding the binding site. A similar effect could also be seen in the fragments between chymotrypsin and trypsin cavities, where the added depth of chymotrypsin S1 cavities, used to bind large hydrophobic side chains, led to significant variations within cubes in that region [61]. Overall, categorically significant fragments generally revealed the same observations as those made with our standard model.

**Figure 9:**
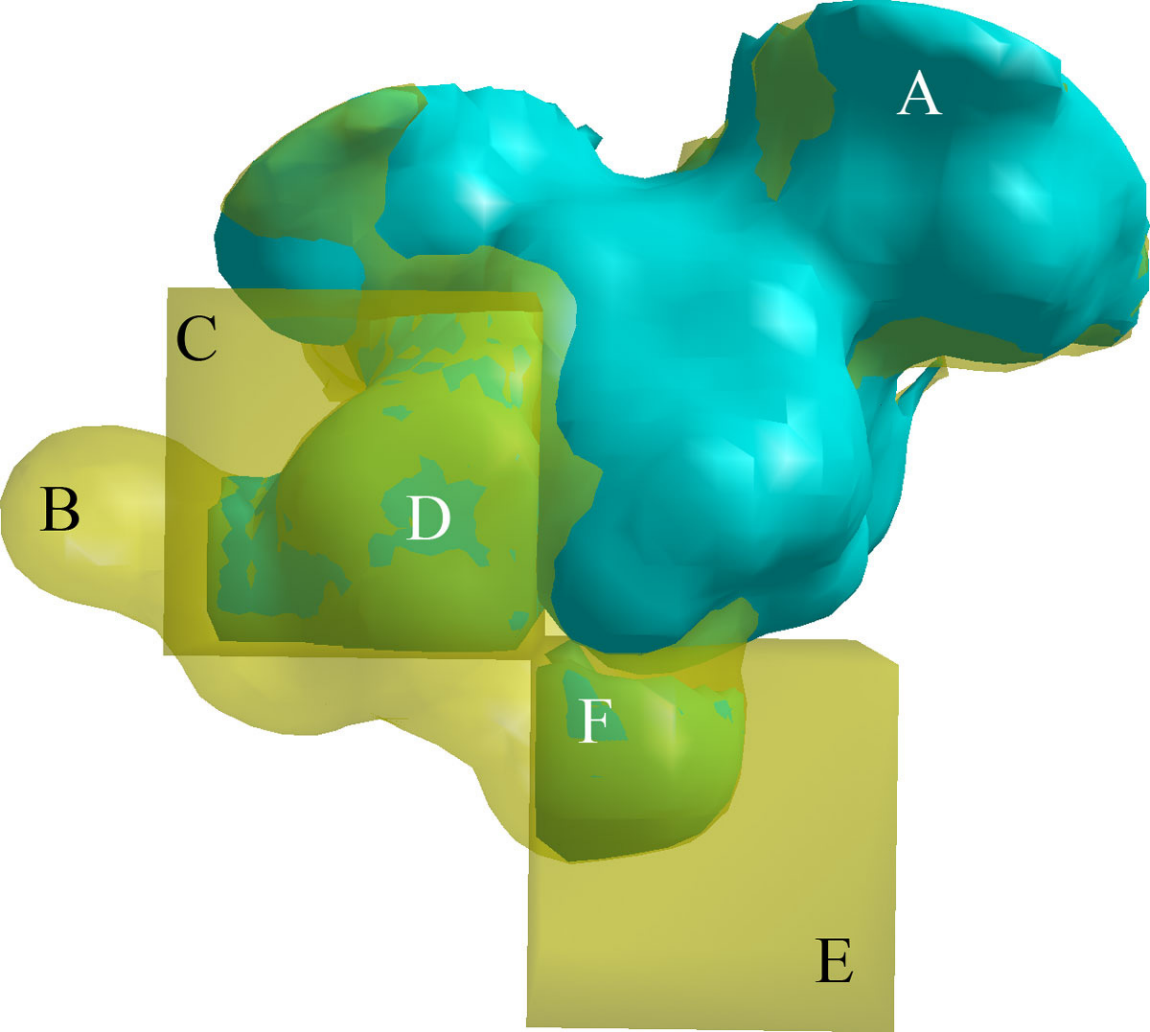
**Fragments in regional models**. A. The S1 subsite of atlantic salmon elastase (pdb: 1elt) shown in teal. B. The S1 subsite of human trypsin (pdb: 1h4w) shown in transparent yellow. C. A user-defined cube, shown in transparent yellow, within which the volume of fragments between the S1 subsites of training set trypsins varies considerably. D. A statistically significant fragment inside C, shown in teal, between the S1 subsites of 1h4w and 1elt, with volume 55 Å^3^. E. A user-defined cube, shown in transparent yellow, within which the volume of fragments between the S1 subsites of training set trypsins varies only a little. F. A statistically significant fragment inside E, shown in teal, between the S1 subsites of 1h4w and 1elt, with volume 17 Å^3^.

Regionalized modeling generated *p*-values that differed considerably from the standard model and from other regions. For example, one 55 Å^3 ^fragment (Figure 9D) in a cube intersecting the S1 subsite of both 1h4w and atlantic salmon elastase (Figure 9C) was assigned the *p*-value .02, while a much smaller 17 Å^3 ^fragment (Figure 9F) in another cube (Figure 9E) received a similar *p*-value. Both fragments are in regions that trypsin requires for recognizing larger amino acids, but the difference in volume indicates that structural variability in trypsins is considerably larger in the first cube relative to the second cube.

## Conclusions

We have presented a computational method for generating fragments and two statistical models for estimating the significance of fragment volume. These methods represent the first algorithms capable of separating individual differences in cavity shape, and also the first to measure their statistical significance, creating a new strategy for identifying influences on protein-ligand binding specificity.

After verifying the choice of distributions for our statistical model, we used our standard model to identify statistically significant fragments between serine protease and enolase cavities. In both cases, the largest fragment between cavities with different binding preferences was always statistically significant, while all fragments between cavities with identical binding preferences were rarely so. By identifying differences in binding cavity shape that are too large to have randomly occurred between cavities with identical binding preferences, this approach predicts cavity regions that drive different binding preferences.

We verified the accuracy of some of these predictions by relating them to experimentally established observations, where possible. In both serine protease and enolase datasets, the most statistically significant fragment was frequently a difference in binding cavity geometry that enabled the accommodation of differently shaped substrates. While other physical phenomena (e.g. electrostatics [62]) are known to influence specificity in both datasets, statistically significant fragments remained strong markers of structural influences on specificity. On other data sets, variations in shape may not be as strongly correlated with specificity. This possibility points to potentials for future work.

Using our regionalized model, we observed that statistically significant fragments in different regions could have very different volumes. These observations indicate that variations between cavities are considerably larger in some regions than in others, and that we can identify such regions. From an applied perspective, this approach could be used to identify regions where small differences in ligand shape could lead to altered or more selective binding, and other regions larger variations in ligand shape do not affect specificity.

## Competing interests

The authors declare that they have no competing interests.

## Authors' contributions

BC concieved of the study. BC and SB developed the statistical model. BC carried out the volumetric analysis. SB trained and validated the statistical model. All authors drafted, read and approved the final manuscript.
